# Intrasubstance Patellar Tendon Repair with the Addition of a Bio-inductive Implant

**DOI:** 10.1016/j.eats.2022.08.051

**Published:** 2022-12-21

**Authors:** Jack T. Bragg, Margaret V. Shields, Matthew J. Salzler

**Affiliations:** From the Department of Orthopaedic Surgery, Tufts Medical Center, Boston Massachusetts, U.S.A.

## Abstract

Intrasubstance patellar tendon ruptures are an uncommon injury that can have devastating long-term effects for patients. Operative intervention to repair the ruptured tendon is the gold standard treatment for these injuries and can be performed using a variety of techniques. Unlike the more common patellar tendon ruptures at the level of the patella, repairs of intrasubstance ruptures are often challenging because of the poor quality of the remaining tendon fibers. Tendon repair with augmentation via bio-inductive implants has gained popularity in upper extremity literature, as it has demonstrated improved tendon strength and patient outcomes. However, there remains a sparsity of reports regarding tendon augmentation in the lower extremity literature. Here, we describe repair of an intrasubstance patellar tendon rupture using a modified SpeedBridge repair and augmentation with a bio-inductive implant.

## Introduction

Intrasubstance patellar tendon ruptures are rare injuries that can be extremely debilitating for patients and require surgery to restore function. Multiple surgical techniques exist for patellar tendon ruptures and have been previously described in the literature, including primary repair with allograft augmentation,^1^ primary repair with suture anchor fixation,^2^ SpeedBridge repair,^3^ and repair with synthetic ligament augmentation.^4^ However, these studies all discuss the repair of healthy tendon tissue and its reattachment to bone, unlike intrasubstance tears that often involve unhealthy tendon tissue and necessitate much more difficult to obtain tendon-to-tendon healing. Bioaugmentation is a new and emerging field that offers a different technique to increase the strength and healing potential of the injured tendon.

Regeneten (Smith & Nephew, Andover, MA) is a bio-inductive implant made of bovine type I collagen from the Achilles tendon. This implant has the capacity to act as a highly porous support that promotes regenerative cell attachment, thereby aiding in tissue healing. In the literature, there are numerous reports of the use of the Regeneten implant in rotator cuff repairs.^5^, ^6^, ^7^, ^8^ However, to our knowledge, literature reports of its utility in lower extremity injuries are limited to three cases of patients suffering from patellar and hamstring tendinopathy^9^^,^^10^ and three technique descriptions for hip abductor repairs and hip capsular repairs without follow up for these patients.^11^, ^12^, ^13^

Here, we present a treatment technique for an intrasubstance patellar tendon tear using a modified SpeedBridge repair, as previously described by Rose et al.,^3^ in addition to augmentation of the repair with the Smith & Nephew Bio-inductive Regeneten implant.

## Surgical Technique

**Fig 1:**
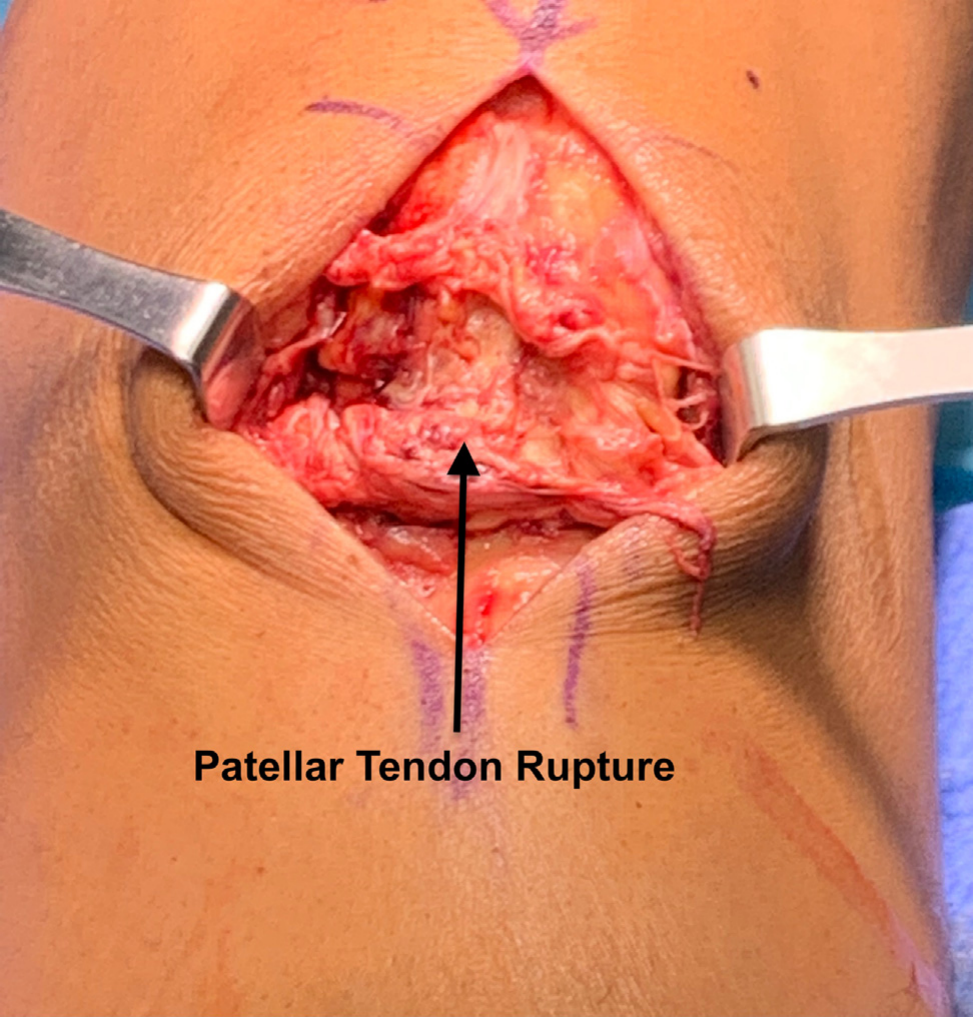
Patient is in the supine position with the proximal tendon stump toward the top of the image and the distal tendon stump toward the bottom. The intrasubstance patellar tendon rupture is easily identified (black arrow) following incision of the paratenon and debridement of scar tissue.

Video 1 provides a narrated overview of the described surgical technique. The patient is positioned supine on a regular operating room table with a nonsterile tourniquet at the thigh level. A straight midline incision is used, extending from the inferior border of the patella to the tibial tubercle. The subcutaneous tissue is dissected, and the paratenon is incised and reflected to expose the patellar tendon. The intrasubstance patellar tendon rupture is identified and debrided of scar tissue to aid in the healing process (Fig 1). Both the proximal and distal attachments of the tendon are inspected to ensure no presence of secondary tears. The medial and lateral recesses are inspected to assess for retinacular tears.

**Fig 2:**
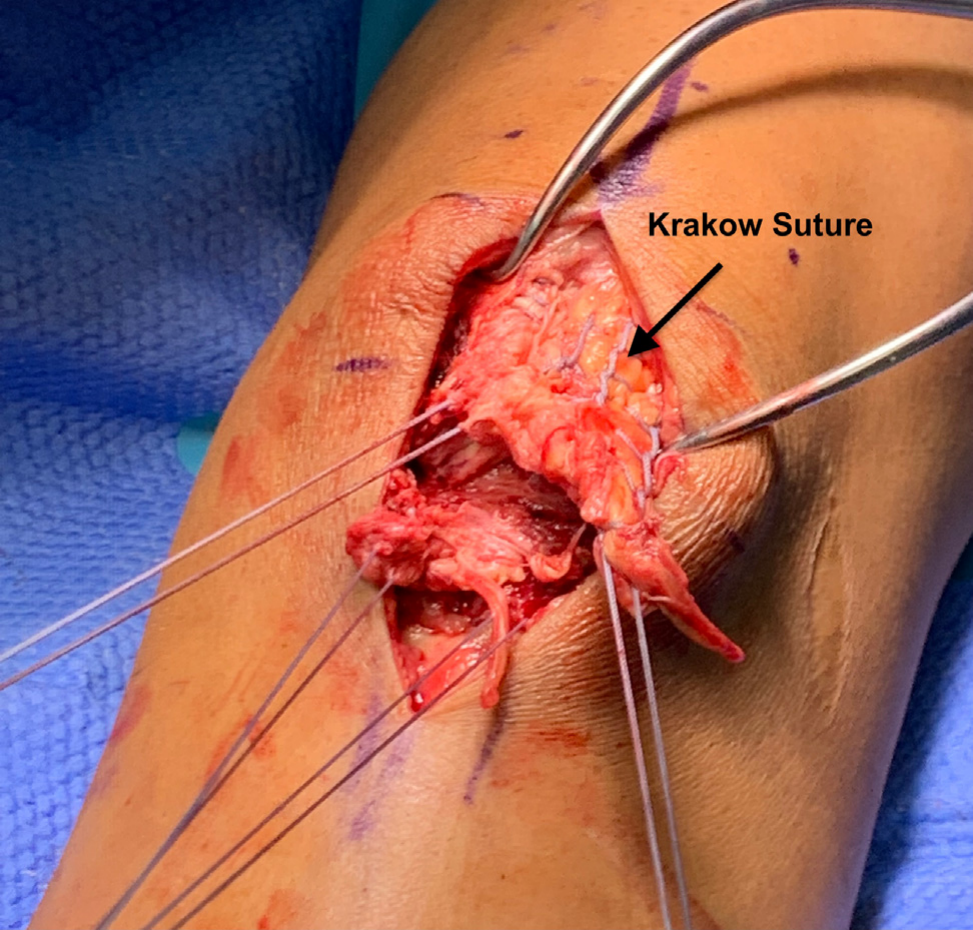
Patient is in the supine position with the proximal tendon stump toward the top of the image and the distal tendon stump toward the bottom. Four equally spaced FiberWires whipstitched in the Krakow fashion with 2 on the distal aspect of the patellar tendon and 2 on the proximal aspect of the tendon.

**Fig 3:**
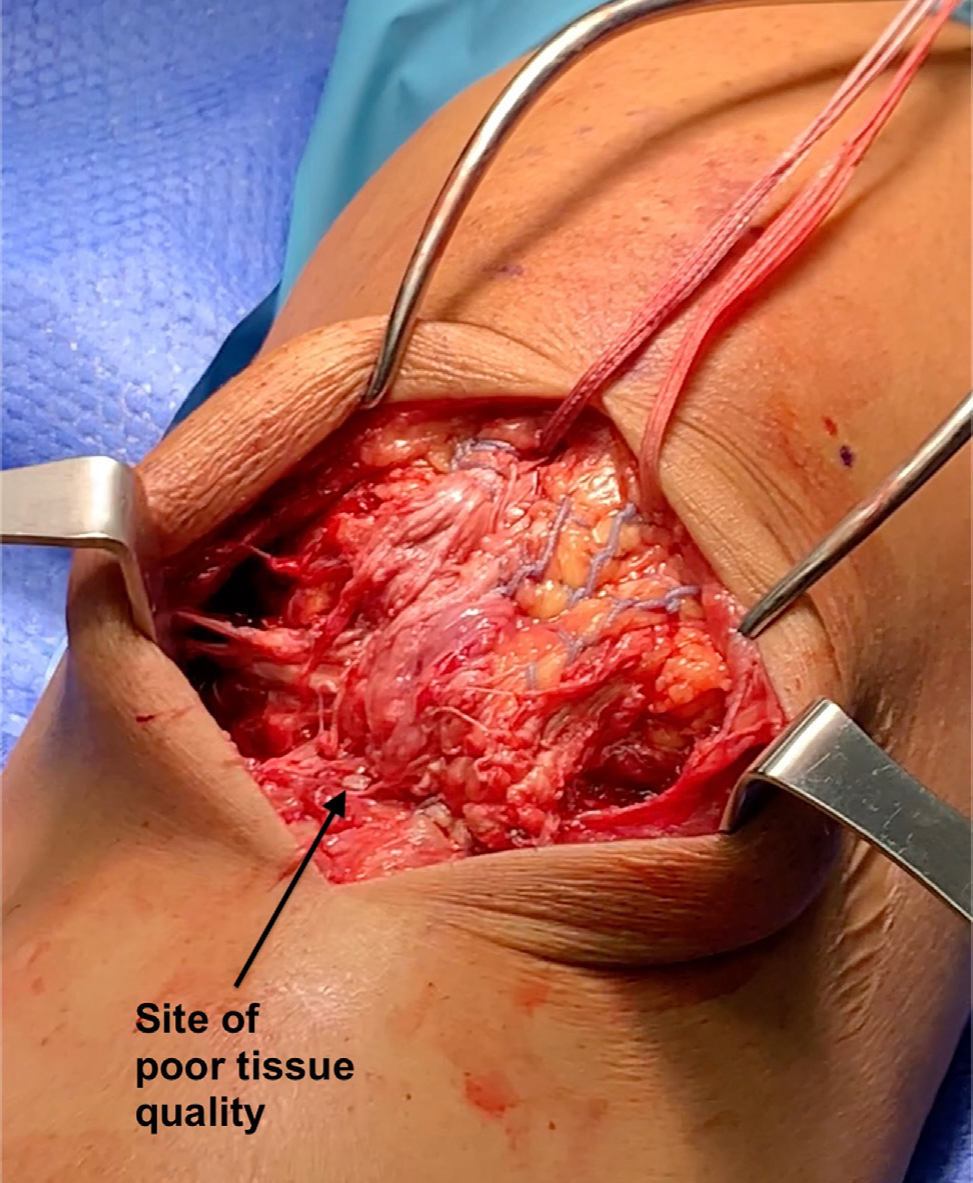
Patient is in the supine position with the proximal limb toward the top of the image and the distal limb toward the bottom. Whipstitched FiberWires are joined to form the primary tendon repair. Visualized in the center of the repair is poor tissue quality (black arrow) that prompted the use of the bio-inductive implant.

After identification and debridement of the tear, attention is turned to the repair. Two #5 FiberWires (Arthrex, Naples FL) are whipstitched in a Krakow fashion on both the proximal and distal ends of the patellar tendon to create four equally spaced sutures (Fig 2). The inferior border of the patella is identified and serves as the proximal site for anchor fixation. Standard technique is used to insert two Arthrex Achilles SpeedBridge Biocomposite SwiveLocks at the inferior border of the patella. The FiberTapes attached to the anchor system are passed through the patellar tendon from deep to superficial at its origin on the distal aspect of the patella on both the lateral and medial aspect of the tendon. The primary tendon repair then takes place with the FiberWires tied under maximal handheld tension in order to rejoin the proximal and distal tendon segments (Fig 3).

**Fig 4:**
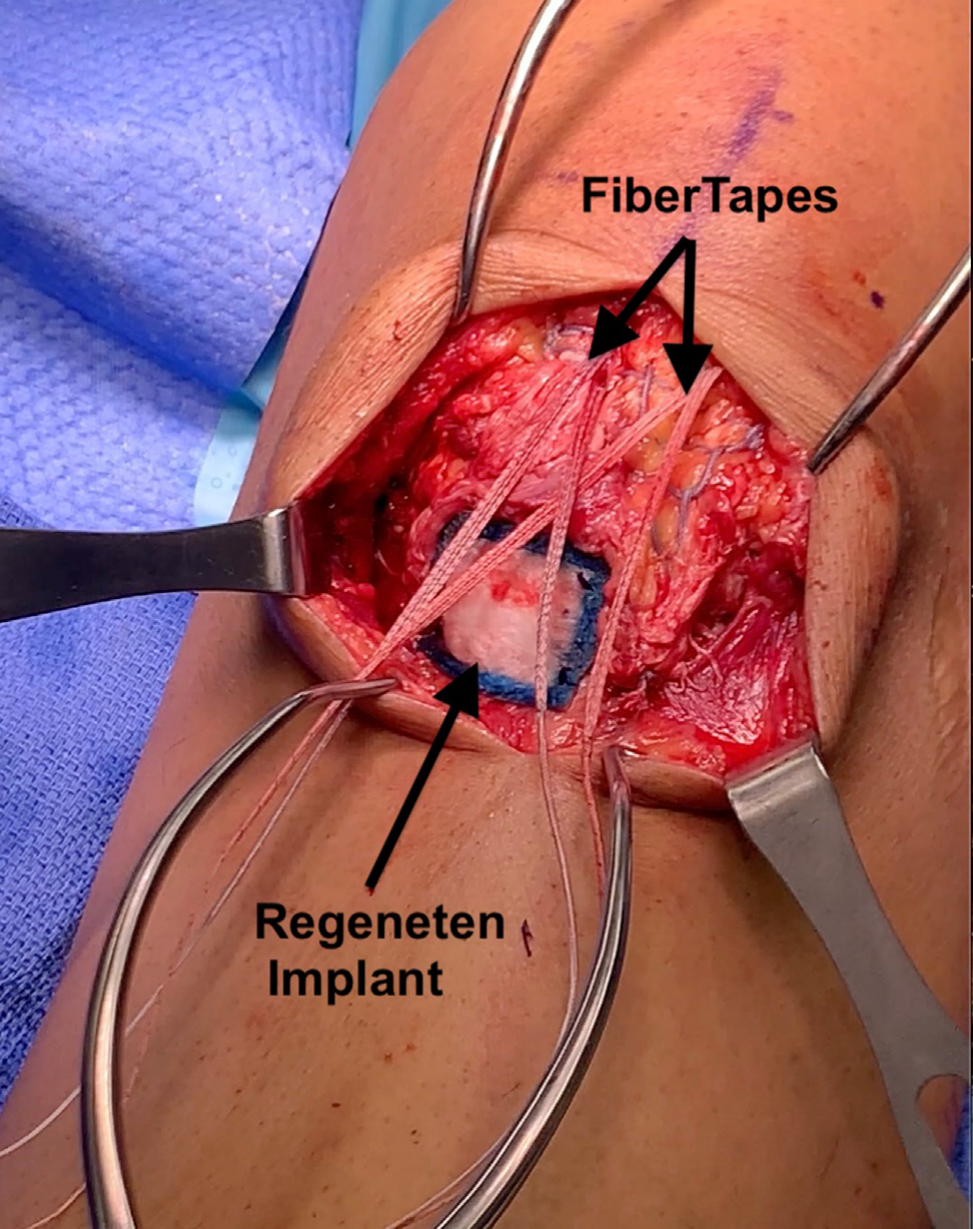
Patient is in the supine position with the proximal limb toward the top of the image and the distal limb toward the bottom. A Regeneten bio-inductive patch is placed over the area of poorest tissue quality on the sutured patellar tendon repair. Additionally visualized are the FiberTapes passing through the patellar tendon from posterior to anterior that will be fixed to the proximal tibia.

Following primary patellar tendon repair and repair of the medial retinacular tear, the Regeneten Bio-inductive Implant is opened on the sterile field. The location of the implant is determined by identifying the poorest tissue quality at the repair site. The implant is deployed over this site and affixed using bioabsorbable staples (Fig 4).

**Fig 5:**
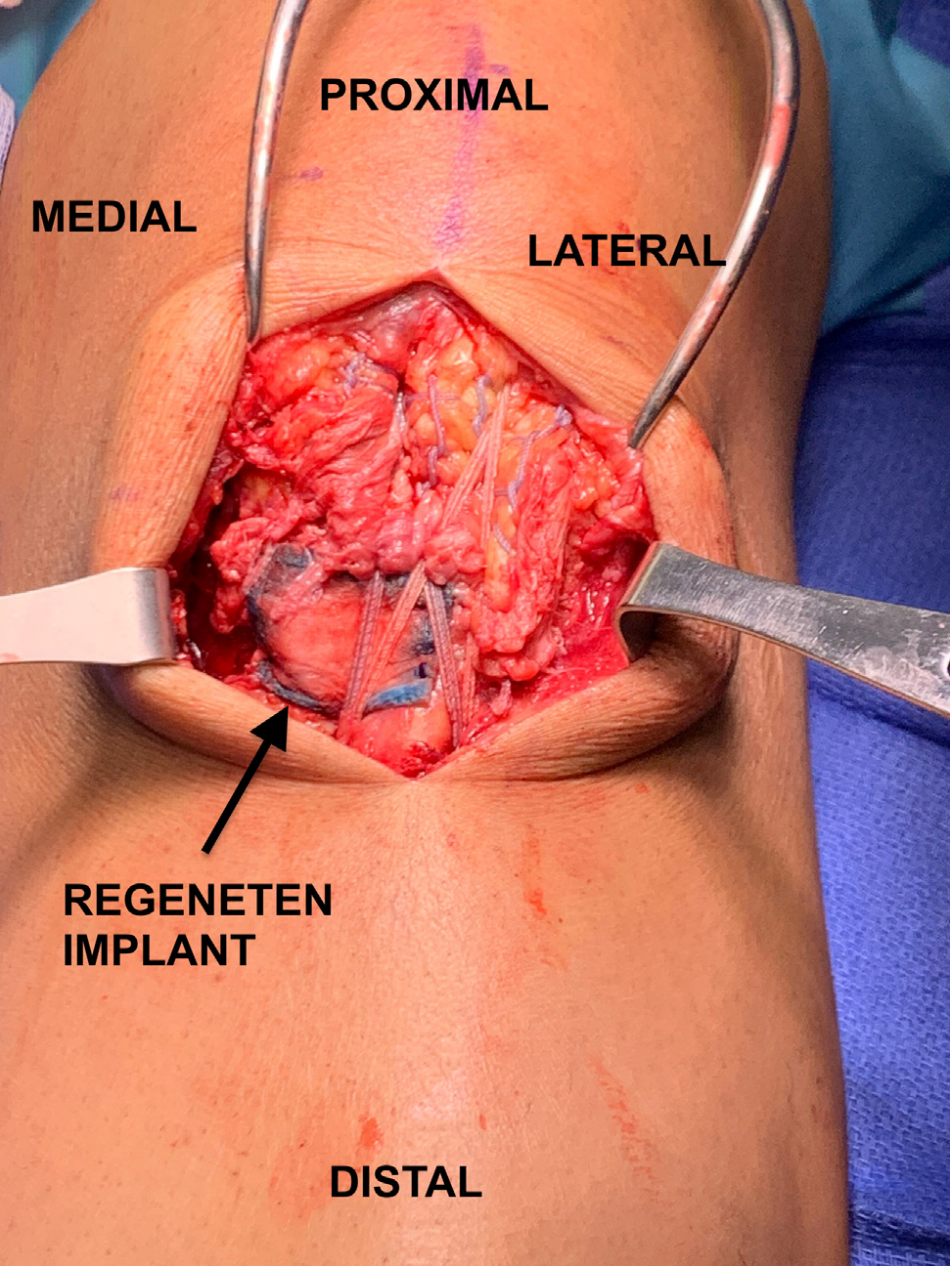
The patient is in the supine position with the orientation as noted. Secured FiberTapes are joined in an intersecting pattern over the Regeneten implant, providing reinforcement of the patellar tendon repair and increased stability of the Regenten patch.

Following deployment of the patch, the distal anchors are placed just medial and lateral to the tibial tubercle using the Arthrex Achilles SpeedBridge Biocomposite SwiveLock system. The intersecting FiberTapes are secured over the patellar tendon and, thus, provide further reinforcement for the primary repair, as well as stability to the position of the bio-inductive implant (Fig 5).

The knee is then taken through a gentle range of motion to establish safe postoperative range-of-motion restrictions and ensure adequate surgical fixation. The extremity is then placed in a hinged knee brace locked in full extension.

Table 1 contains a summary of the pearls and pitfalls of this technique.

**Table 1:** Pearls and Pitfalls of This Technique

Pearls	Pitfalls
Bio-inductive implant should be placed in an area of poorest tissue quality.	Ensure adequate fixation of the implant to the tissue with bioabsorbable staples.
The Arthrex Achilles SpeedBridge Biocomposite SwiveLock system can be used superficial to the bio-inductive implant for additional support of the implant and additional strength of tendon repair.	
Krakow sutures should be tied under maximal tension to ensure appropriate closure of tendon defect.	

### Rehabilitation

Postoperative rehabilitation for the patellar tendon repair with Regeneten augmentation follows that of standard patellar tendon repair protocol. A hinged brace remains locked at full extension for 6 weeks, and during this time, the patient is allowed to bear weight as tolerated. Physical therapy is initiated 10-14 days postoperatively and is focused on regaining strength and motion. At 6 weeks postoperatively, flexion is gradually increased at 30° every week. The brace is then discontinued at 10 weeks postoperatively, if there is adequate quadriceps strength.

## Discussion

Intrasubstance patellar tendon ruptures are an uncommon phenomenon that require surgical repair in order to restore patient function. A retrospective study recently found that suture anchor repair techniques, a version of which is described here, leads to lower rates of rerupture compared to transosseous repair techniques for patellar tendon ruptures from the inferior pole of patella or tibia tubercle.^14^ However, intrasubstance tears are unique in that stimulating tendon-to-tendon healing is much more difficult than stimulating tendon to bone healing, which is more commonly seen with patellar tendon ruptures. The addition of a bio-inductive patch as described in this technique has the ability to improve the quality of the repair by augmenting the biology of the tissue surrounding the repair, thus enhancing the healing potential. Although the patch does not increase the strength of the tendon repair directly, prior studies of patients with rotator cuff tears have shown that the addition of a bio-inductive patch results in improved functional and pain outcomes, as well as demonstrating significantly increased tendon thickness when evaluated on an MRI.^7^ Additionally, other studies have demonstrated that there is no evidence of an inflammatory or foreign body reaction in the tissue, a concern when implanting any foreign material and can delay healing.^15^ Although the use of a bio-inductive patch has been shown in biological studies to improve healing potential of tendons, there remains a sparsity of literature regarding the use of this implant in lower extremity pathologies. To our knowledge, there have only been a few reported uses of the bio-inductive implant in cases of patellar and hamstring tendinopathy,^9^^,^^10^ hip abductor repair,^11^^,^^12^ and hip capsular repair^13^ with no reports of its use in complete patellar tendon rupture, as reported here. In the cases that had follow-up for their patients, there was noted a decrease in pain, a quicker time to recovery, and increased tendon thickness visualized on MRI with use of the bio-inductive patch.^9^^,^^10^ With promising benefits reported in biological studies and upper extremity literature, we anticipate increased use of tendon augmentation with the bio-inductive patch in the future. This article describes our preferred technique for this novel treatment of intrasubstance patellar tendon rupture.

While the potential benefits are described previously, there are also limitations of this technique. The cost of the bio-inductive implants must be considered as the use of such an implant has been shown in the upper extremity literature to be more costly than performing a traditional repair technique, which we imagine would hold true with surgical repair of the patellar tendon.^16^ Given that this is a new technique, rerupture rates have not been established, but studies have demonstrated that the use of these implants is safe and that they can promote new tendon formation months after surgery with some suggestions of improved healing rate.^15^^,^^17^ Further studies are needed to investigate the long-term outcomes of patients with patellar tendon tears that are repaired using augmentation with bio-inductive implants.

A summary of the advantages and disadvantages of our technique can be found in Table 2.

**Table 2:** Advantages and Disadvantages of This Technique

Advantages	Disadvantages
Improved tendon healing potential with the use of the bio-inductive implant	Increased cost associated with the use of the bio-inductive implant
	Slightly increased surgical time due to deployment and fixation of implant

## Supplementary Data

ICMJE author disclosure forms

Video 1 The patient is placed in the supine position. After incising the paratenon, the patellar tendon rupture is easily identified. Following debridement of scar tissue at the ruptured tendon edges, FiberWire is used to create four equally spaced Krakow sutures, two on the distal tendon stump and two on the proximal tendon stump. A SpeedBridge anchor is placed into the inferior border of the patella, and the FiberTapes are advanced through the tendon. The free FiberWire ends are then brought together and tied under maximal handheld tension. The site of poorest tissue quality is identified, and the Regeneten implant is deployed and stapled into position. To complete the repair, the FiberTapes from the SpeedBridge anchors are secured distally at the medial and lateral border of the tibial tubercle.
